# Single Cell Sequencing of the Pineal Gland: The Next Chapter

**DOI:** 10.3389/fendo.2019.00590

**Published:** 2019-09-20

**Authors:** Steven L. Coon, Cong Fu, Steven W. Hartley, Lynne Holtzclaw, Joseph C. Mays, Michael C. Kelly, Matthew W. Kelley, James C. Mullikin, Martin F. Rath, Luis E. Savastano, David C. Klein

**Affiliations:** ^1^Molecular Genomics Core, Office of the Scientific Director, Eunice Kennedy Shriver National Institute of Child Health and Human Development, National Institutes of Health, Bethesda, MD, United States; ^2^Key Laboratory of Organ Regeneration & Transplantation of the Ministry of Education, The First Hospital of Jilin University, Changchun, China; ^3^National-Local Joint Engineering Laboratory of Animal Models for Human Diseases, Changchun, China; ^4^Comparative Genomics Analysis Unit, Cancer Genetics and Comparative Genomics Branch, National Human Genome Research Institute, National Institutes of Health, Bethesda, MD, United States; ^5^Microscopy and Imaging Core, Office of the Scientific Director, Intramural Research Program, Eunice Kennedy Shriver National Institute of Child Health and Human Development, National Institutes of Health, Bethesda, MD, United States; ^6^Institute on Systems Genetics, New York University School of Medicine, New York, NY, United States; ^7^Single Cell Analysis Facility, Frederick National Lab for Cancer Research, National Cancer Institute, National Institutes of Health, Bethesda, MD, United States; ^8^Section on Developmental Neuroscience, Laboratory of Cochlear Development, Division of Intramural Research, National Institute on Deafness and Other Communication Disorders, National Institutes of Health, Bethesda, MD, United States; ^9^National Institutes of Health Intramural Sequencing Center, National Human Genome Research Institute, National Institutes of Health, Rockville, MD, United States; ^10^Department of Neuroscience, Panum Institute, University of Copenhagen, Copenhagen, Denmark; ^11^Department of Neurosurgery, University of Michigan, Ann Arbor, MI, United States; ^12^Office of the Scientific Director, Intramural Research Program, Eunice Kennedy Shriver National Institute of Child Health and Human Development, National Institutes of Health, Bethesda, MD, United States

**Keywords:** pineal, single cell sequencing, melatonin, transcriptomics, adrenergic, transcriptome profiling

## Abstract

The analysis of pineal cell biology has undergone remarkable development as techniques have become available which allow for sequencing of entire transcriptomes and, most recently, the sequencing of the transcriptome of individual cells. Identification of at least nine distinct cell types in the rat pineal gland has been made possible, allowing identification of the precise cells of origin and expression of transcripts for the first time. Here the history and current state of knowledge generated by these transcriptomic efforts is reviewed, with emphasis on the insights suggested by the findings.

## Introduction

The pineal gland is composed 90–95% of pinealocytes, which synthesize melatonin (1). Studies of the pineal gland have addressed the levels of transcripts involved in this process and have experienced remarkable improvements, innovations, and enhancements, in parallel with advances in cell biological techniques that have characterized the field. In general, genes expressed exclusively in non-pinealocytes have been ignored.

The first efforts to study a single mRNA transcript in the pineal gland came from northern blot analysis in the late 1980s (2–5). It required the equivalent of several rat pineal glands (4–5 mg wet weight); RNA was extracted, electrophoresed and blotted. This allowed for the radiochemical detection of transcripts encoding tryptophan hydroxylase1 (*Tph1*) and acetylserotonin methyltransferase (*Asmt)*/hydroxyindole-*O-*methyltransferase, the transcripts that encode the first and last enzymes in melatonin synthesis, respectively. The northern blot technique was highly useful, especially because it allowed the resolution of distinct molecular species. However, it was obviously limited by the amount of tissue required and the small number of transcripts it could detect on repeated stripping and probing of blots.

The reverse transcription polymerase chain reaction was introduced into the pineal literature early in the 1990s (6–13). It was highly popular because it was sensitive and allowed multiple transcripts to be measured using small amounts of mRNA. It was used to detect low levels of transcripts including receptors and clock genes. However, quantitation with the method was somewhat unreliable and results could only reflect changes in small regions of mRNA amplified by the technique, which permits off-target results and precludes examination of the entire transcripts, which may have reflected gene leakage. Another problem with PCR was overamplification of very weakly expressed transcripts. In addition, analysis of each transcript was hands-on, limiting the number of transcripts that could be detected on a routine basis.

A revolutionary method was introduced to pineal cell biology with cDNA arrays, which at the start allowed for the detection of several hundred targets (14) and ultimately developed into microarrays, which permitted thousands of targets to be probed simultaneously using as little as one rat pineal gland (15–19) or 10 larval zebrafish pineal glands (20). However, this technique had the disadvantage of probing only portions of a transcript and was only useful for those transcripts which were represented on the microarray chip. Putting aside these limitations, this technique made important advances by reducing the amount of tissue required and increasing the number of genes probed. In the case of the rat pineal gland, it revealed large day/night changes in hundreds of transcripts, many more than had been realized at the time (19). The technique was also useful in comparing the pineal gland and retina and in determining the large number of genes shared by these two tissues.

The limitations of the cDNA chip technology were rapidly overcome in the early years of this century with the development of methods that sequenced the entire transcriptome, also referred to as bulk sequencing. Sequences have been obtained for chicken, human, mouse, rat, rhesus, and zebrafish pineal glands (21–30). This provided the sequence of full length transcripts, including the coding and flanking regions. It also provided an indication of splicing and alternative polyA sites. It sequenced all transcripts, known and unknown, including noncoding long and short RNAs (21, 29, 31). The technique is remarkably sensitive, allowing for tens of thousands of transcripts to be sequenced with the mRNA from a fraction of a single rat pineal gland.

**Figure 1:**
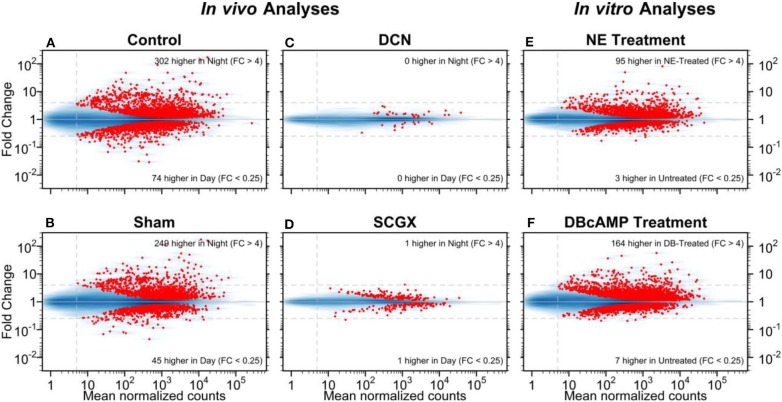
Day/night differences in gene expression. The mean normalized read-pair counts (x-axis) vs. the estimated fold change (y-axis) are displayed on a log-log scale. Four *in vivo* and two *in vitro* analyses are presented as MA plots. The blue shading indicates the density of genes, and each red point represents a gene with statistically significant differential expression (adjusted-*p* < 0.001). Dashed horizontal lines mark 4-fold changes in both directions; the dashed vertical line indicates the minimum abundance threshold for the statistical tests. The four *in vivo* analyses compared night and day time points in adult rats for the following groups: **(A)** no surgery (Control); **(B)** neonatal sham surgery (Sham); **(C)** neonatal superior cervical ganglia decentralization (DCN); **(D)** neonatal superior cervical ganglionectomy (SCGX). The two *in vitro* analyses compared treated/untreated pineal glands: **(E)** norepinephrine-treated (NE) vs. untreated and **(F)** dibutyryl-cyclic-AMP-treated (DBcAMP) vs untreated. Reproduced from Hartley et al. (25). This figure and associated legend is published with permission of the original publisher under license CC0.1.0.

As applied to the pineal gland, this technique provided excellent data on day/night differences. Moreover, studies on the rat pineal gland have provided valuable information on the effects of superior cervical ganglionectomy (SCGX) or decentralization (DCN) in *in vivo* experiments, and the effects of norepinephrine or dibutyryl cyclic AMP in *in vitro* experiments (Figure 1) (25). These confirmed and expanded previous results on the rat pineal gland, which showed that there was a broad change in the transcriptome on a 24-h basis. It also showed that neural stimulation of this tissue, in the form of postganglionic projections from the superior cervical ganglia stimulated the gland, based on the observation that both forms of surgical denervation, SCGX and DCN, blocked these changes. In addition, it revealed that most of these changes could be driven *in vitro* by norepinephrine or by its second messenger cyclic AMP. It is noteworthy that comparison of the transcripts that were induced more than 4-fold at night and by norepinephrine or dibutyryl cyclic AMP were nearly identical, numbering about 50 [Table S1 in (25)]. This correlation supported the view that the day/night differences were driven by a norepinephrine-cyclic AMP mechanism. It should be noted that the correlation was lower with weakly induced genes, which may be a reflection of statistical variation.

The development of advanced sequencing methods has evolved and deserves brief mention here. A hybrid approach is now available that combines Illumina short-read/high-throughput RNA-Seq with targeted qPCR and long-read Pacific Biosciences SMRT sequencing. In pineal gland studies it has been possible to identify 20 alternative RNA isoforms of the Ttc8/BBS8 gene (23). This gene was known to exist in multiple isoforms and is of interest because of evidence that it is involved in the Bardet-Biedl syndrome and non-syndromic retinitis pigmentosa (32–35). This technique is severely limited by the number of genes it can detect on a practical basis, but holds great promise for the study of isoforms, a complex and difficult endeavor. The interested reader is referred to the original publication for more details (23).

The most recent advance in sequencing is single cell RNA sequencing (scRNA-seq) (36). It takes several forms all of which allow for thousands of single cells to be sequenced simultaneously, yielding several thousand transcripts per cell. Overall, the technique has extremely high sensitivity and generates an enormous amount of data on the transcriptomes expressed in individual cells.

The technique was introduced into the pineal literature because of the suggestion that there were two populations of the cell that were defined by large differences in ASMT protein (37). As mentioned above, ASMT is the last enzyme in melatonin synthesis and converts the melatonin precursor N-acetylserotonin to melatonin. We hoped that the new technology would provide a transcriptional profile of each cell type and answer the question of whether pinealocyte subtypes defined by different levels of ASMT exist.

## Single Cell RNA Sequencing

### Cell Isolation

The isolation of single pineal cells followed a well-established method, which has been used for biochemical, electrophysiological, and cytochemical studies (38–42). Glands were removed, soaked in DMEM solution and then cleaned under a microscope to limit the contaminating cells coming from blood and connective tissue. The glands were then placed in a freshly prepared Papain Dissociation System (Worthington; Lakewood, NJ) containing DNAase; details of the procedure have been published (43).

### Single Cell Analysis

Single-cell cDNA libraries were constructed using a Chromium Controller (10X Genomics; Pleasanton, CA) and the Chromium Single Cell 3′ Reagent Kits v2 (43). In brief, dilute solutions of completely dissociated preparations of single cells were introduced into a stream of oil to make microdroplets. Sequencing reagents in the stream included a “sponge” that contains a unique cDNA marker for identification of the cell source of each transcript: one marker—one cell. This unique cDNA marker was incorporated into the mRNA at the polyA end, thereby providing a means of tracking the originating cell source of each molecule.

The microdroplets containing individual cells were mixed together and sequenced (Illumina HiSeq2500, Illumina; San Diego, CA). Ninety-eight bp sequences were produced in close proximity to the polyA tails. It was possible to recover 2,400–4,300 cells per sample, with 40–70 k reads per cell and 2,700–3,000 genes per cell detected on average (43).

The analysis of sequenced single-cell libraries was done by generating gene-level counts with the CellRanger analysis software v2.1.0 (10X Genomics). This aligns sequencing reads to the rat Rnor6.0 reference genome (Ensembl). The sequenced cells were subsequently filtered to remove doublets and low abundance genes. Dimensional reduction analysis was done (Seurat v2.2.0 package for R). Gene counts were normalized to 10^4^ molecules per cell. Lists of ~1,500 highly variable genes for the day and the night samples were prepared and used to compute principal components (PC) using RunPCA; the results of PC analysis were projected onto the remaining genes with ProjectPCA (43).

The clustering of cells was done by employing a shared nearest neighbor (SNN)-based algorithm; results were imaged by t-distributed stochastic neighbor embedding (t-SNE) through RunTSNE (parameters: do.fast = TRUE). The 2D projections of the cells generated by this method generates clusters that are color-coded according to FindClusters output. The identity of the clusters were determined using known marker genes. In each sample, the β-pinealocyte population was embedded on the t-SNE plot as a single cluster; it was divided into smaller color-coded clusters by the SNN clustering algorithm. These clusters were consolidated into one large cluster for subsequent study to match the t-SNE embedding. Cellular doublets were eliminated based on expression of moderate-to-high levels of genes that were markers for separate clusters (day, *n* = 60; night, *n* = 125) (see reference (43) for additional details).

## Nine Cell Types of the Pineal Gland

**Figure 2:**
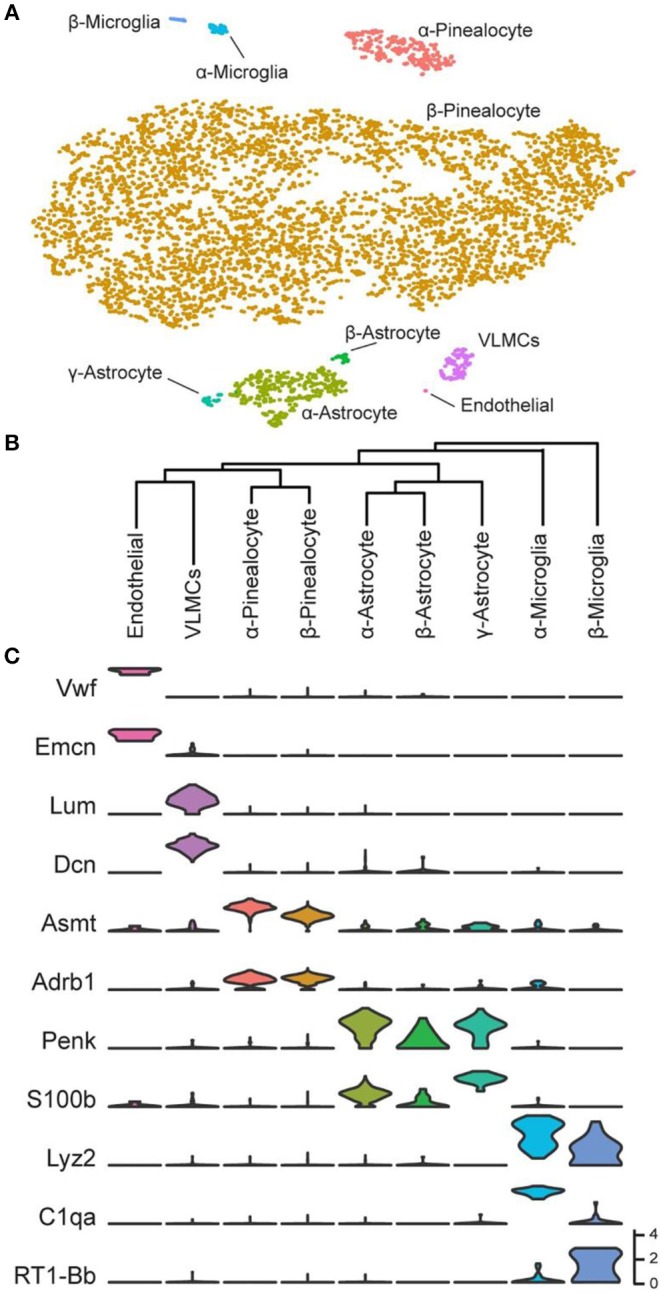
Transcriptomic characterization of cell types in the daytime rat pineal gland. **(A)** t-Distributed stochastic neighbor embedding (t-SNE) visualization of over 5,000 daytime rat pineal gland cells as profiled by scRNA-seq. Cell types are color-coded by cluster. **(B)** Hierarchical clustering dendrogram showing cell type transcriptomic similarity, including two pinealocyte subtypes, the three astrocyte subtypes, the two microglia subtypes, and two vascular-associated cell types: VLMCs and endothelial cells. **(C)** Violin plots of marker genes for cells from each cell type. Y-Axis is natural log of normalized counts. Reproduced from Mays et al. (43). This figure and associated legend is published with permission of the original publisher under license CC0.1.0.

Over 5,000 individual cells were subjected to cluster analysis, which detected five major cell types: pinealocytes, astrocytes, microglia, vascular and leptomeningeal cells (VLMCs), and endothelial cells (Figure 2A). The expression of marker genes in these cells confirmed this finding. It was possible to further resolve the cell types according to the results of cluster analysis and marker gene abundance into two pinealocyte subypes (α and β), three subtypes of astrocytes (α, β, and γ), and two subtypes of microglia (α and β). Transcriptomic relationships of the nine cell types as indicated by hierarchical clustering are consistent with our assigned designations (Figure 2B).

### Pinealocytes

**Figure 3:**
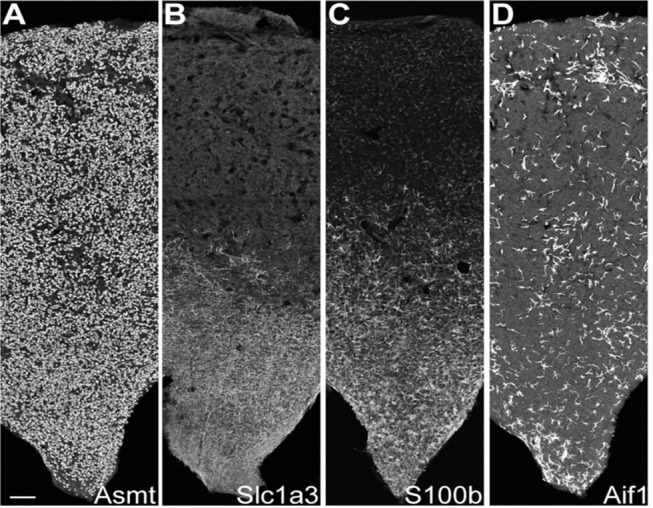
IHC reveals cell type-specific patterns of expression. IHC sections through the rat pineal gland midline; rostral stalk origin at the bottom. The length and middle third of the width of the gland appear. Scale bar = 100 μm. **(A)** Uniform distribution of ASMT-positive pinealocytes. **(B)** Slc1a3-positive γ-astrocytes abundance is greatest in rostral/stalk region. **(C)** S100b-positive cells are abundant in the rostral region; they appear elsewhere with distinctly lower density and weaker expression strength. **(D)** Aif1-positive cells are unevenly distributed throughout pineal gland at low density. See Figure S6 in Mays et al. (43) for full images and further details. Reproduced from Mays et al. (43). This figure and associated legend is published with permission of the original publisher under license CC0.1.0.

The precise proportion of cell-types is difficult to determine with a high degree of confidence because of differences in recovery and cell stability during isolation. However, with this limitation in mind, it appears that 90% of the profiled cells were pinealocytes [Table S1 in (43)], which is generally in line with morphological studies (1, 44) (Figure 3A). These cells all expressed high levels of *Tph1, Asmt*, and *Sag* [Figure 2C; Figure S1 in (43)]. These cells also expressed high levels of *Gngt1, Gngt2, Rom1, Crx, Cngb1, Cnga1, Pde6c*, and *Slc6a6;* receptors for adrenergic agonists *Adrb1, Adra1b*, and *Drd4;* and, receptors for cholinergic agonists *Chrna3* and *Chrnb4* [Figure 2C; Figures S1, S2 in (43)]. In addition, these cells expressed a group of 49 transcripts found nearly exclusively in the pineal gland and retina (19) including *Sag* [Figure S1 in (43)], *Gngt1* and *Gngt2* [Figure S4 in (43)], *Crx* and *Neurod1* [Figure S19 in (43)], *Pde6b* [Figure S15 in (43)], *Drd4* [Figure S2 in (43)], and *Cacna1f*, *Cnga1*, and *Cngb1* [Figure S13 in (43)]. The expression of these transcripts exclusively in pinealocytes has not been directly demonstrated previously in most cases; this is because a homogenized mixture of cells in the pineal gland had been used in earlier bulk sequencing studies, precluding the clear association of a gene with a cell type. However, several lines of evidence in the literature point to this conclusion (43).

**Figure 4:**
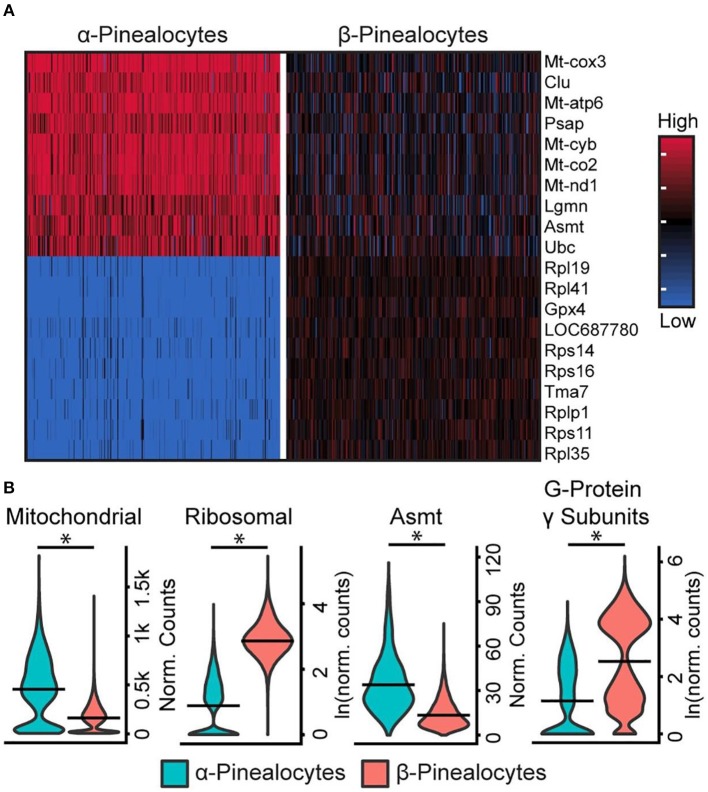
scRNA-seq reveals two transcriptionally distinct pinealocyte populations. **(A)** A heatmap of expression values for the top 10 most differentially expressed genes (by effect size) for α- and β-pinealocytes. Values are Z-scores of counts calculated between all cells of both cell types. Each column represents one cell: random samples of 250 cells per cell type are presented. **(B)** Violin plots of expression distribution differences between two pinealocyte subtypes for three functional groups and one gene, *Asmt*. Y-Axis is either normalized counts or natural log (ln) of normalized counts. Horizontal lines represent the mean. *p < 0.001, Wilcoxon rank sum test. All cells from each subtype are included (α-pinealocyte, n = 275; β-pinealocyte, *n* = 4,822). Mitochondrial group includes differentially expressed mitochondrial OxPhos genes (*p* < 0.05, *N* = 12, fold change ≥2.0), ribosomal group includes top 20 most differential ribosomal genes by effect size (*p* < 0.05, fold change ≥2.0), G-protein γ-subunits include *Gngt1, Gngt2, Gng10*, and *Gng13* [see Figure S5 in (43) for individual genes]. Reproduced from Mays et al. (43). This figure and associated legend is published with permission of the original publisher under license CC0.1.0.

Five percent of pinealocytes were the α-subtype; the remaining pinealocytes were β-subtypes. Although these two cell types share a characteristic set of marker genes and function as sources of melatonin, analysis revealed some distinct genetic differences and differences in sets of functional groups. The most outstanding included (1) *Asmt;* (2) mitochondrial oxidative phosphorylation (OxPhos) genes; (3) genes that comprise the ribosomal genome; and, (4) G-protein γ-subunits [Figure 4A; Figures S1, S3, S4 in (43)]. *Asmt* expression (Figure 4B) in α-pinealocytes was 3.4-fold greater, supporting results from previous immunohistochemical studies of ASMT protein (37). The counts for OxPhos and ribosomal transcriptomes were pooled [Figure S5 in (43)] for analysis; α-pinealocytes had a 2.3-fold greater average expression of eight differentially expressed OxPhos genes, and 8.2-fold lower expression of the top 20 differentially expressed ribosomal genes. There also is a 5.4-fold lower average expression of G-protein γ-subunits *Gngt1, Gngt2, Gngt10*, and *Gng13* in α-pinealocytes relative to β-pinealocytes [Figure 4B; Figure S4 in (43)]. The possibility that α-pinealocytes represent stressed cells was rejected because of the opposite and robust differences between the levels of OxPhos and ribosomal genes: the former being higher in α-pinealocytes and the latter higher in β-pinealocytes.

### Astrocytes

**Figure 5:**
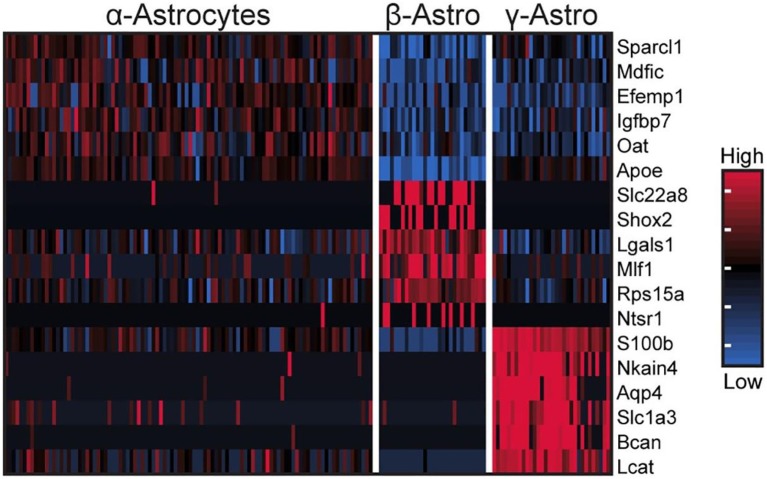
scRNA-seq reveals three transcriptionally distinct astrocyte populations. Heatmap of expression values for the top 6 highest differentially expressed genes (by effect size) for α-, β-, and γ-astrocytes. Values are Z-scores of counts calculated between all cells of the three cell types. Each column represents one cell; a random sample of 100 cells from α-astrocytes are shown; all β- and γ-astrocytes are shown. See also Figure S1 in Mays et al. (43) for more information. Reproduced from Mays et al. (43). This figure and associated legend is published with permission of the original publisher under license CC0.1.0.

These cells accounted for seven percent of the cells [Table S1 in (43)], based on expression of known markers including *Aldh1a1, S100b*, and *Tnfrsf21* [Figure 2C; Figure S1 in (43)] (45–47). These cells also highly expressed *Penk, Apoe*, and *Esm1* [Figure 2C; Figure S1 in (43)]. The percentages of α-, β-, and γ-astrocytes were 85, 7, and 8%, respectively. α-Astrocytes had higher *Sparcl1, Mdfic, Efemp1, Oat*, and *Gad2* expression relative to other subtypes. The β-astrocytes expressed *Slc22a8, Shox2, Lgals1*, and *Mlf1* at higher levels than other subtypes. γ-Astrocytes were characterized by stronger expression of *S100b, Nkain4, Aqp4, Slc1a3, Bcan*, and *Gfap* [Figure 5; Figure S1 in (43)]. Histochemical analysis revealed that γ-astrocytes were primarily limited in distribution to the pineal stalk region, as indicated by Slc1a3 [Figure 3B; Figure S6B (43)], as was true of Gfap protein [Figures S6C, S7C in (43)]; this is consistent with previous observations (48–50). *S100b* was expressed in all astrocyte subtypes, but was strongest in γ-astrocytes [Figure 5; Figure S1 in (43)]. Detection of S100b-postive cells by IHC revealed astrocytes occur throughout the gland, though higher expression was present in the pineal stalk region, consistent with the higher expression of *S100b* exhibited by γ-astrocytes [Figure 3C; Figures S6D, S7D in (43)].

### Microglia

One percent of the profiled cells [Table S1 in (43)] were classified as microglia according to expression of *Aif1* and *Lyz2* [Figure 2C; Figure S8 in (43)] (45–47). AIF1 IHC Positive cells were present throughout the gland [Figure 3D; Figure S6E in (43)]. α- and β-microglia subtypes comprised 64 and 36% of microglia, respectively. These cells were strongly differentiated by complement components *C1qa, C1qb*, and *C1qc*, which were high in α-microglia. β-Microglia in contrast had low levels of the complement component transcripts, but high levels of MHC Class II transcripts *RT1-Da, RT1-Db1*, and *RT1-Ba* [Figure S8 in (43)].

### Vascular Cells

Endothelial cells and VLMCs were detected in low abundance. These cells appear to be in intimate contact, based on expression of *Cdh11 and Gja1* in both [Figure S10 in (43)]. Endothelial cells accounted for 0.1% of cells profiled and were characterized by the expression of *Vwf, Emcn*, and other markers (43).

VLMCs were 2% of the profiled cells, identified by expression markers *Lum, Dcn, Col1a1*, and *Gjb2* [Figure 2C; Figure S9 in (43)] (51). VLMCs have never been described in the pineal gland prior to this study. They have the potential of major importance in acting as mediators between circulating signals and pineal cells, in addition to contributing to the extracellular matrix reflecting the expression of collagen and extracellular matrix proteins.

For example, they have receptors for circulating ligands which could act to alter the synthesis and release of secondary signals that impact the function of other cells in the gland. Pineal VLMCs exclusively express *Il13ra2* [Figure S9 in (43)], the transcript that encodes a selective receptor for the cytokine interleukin Il13 (52). Interaction of Il13 with its receptor could impact the pineal gland broadly, perhaps through effects on the extracellular matrix. In addition, vascular cells could act on pinealocytes and astroctyes through contact-dependent ephrin ligand-receptor mechanisms (53). Specifically, the ephrin ligand EFNA1 on endothelial cells could bind to the ephrin receptor EPHA4 on pinealocytes; and, the ligand EFNB1 on VLMCs could bind to the receptor EPHB1 on astrocytes (Figure S17 in (43)]. It should also be mentioned that, VLMCs are a standout among cells in the pineal gland for expression of the α_2A_-adrenergic receptor, encoded by *Adra2a* [Figure S2 in (43)]. Activation of this receptor by circulating norepinephrine or epinephrine can cause catecholamine-induced inhibition of adenylate cyclase and as a result inhibit cyclic AMP dependent processes in the VLMCs. Although of interest, it should be noted that the above are speculations and accordingly require further study to determine their relevance in the context of pineal cell biology.

## Day and Night Changes in the Pineal Cell Transcriptome

**Figure 6:**
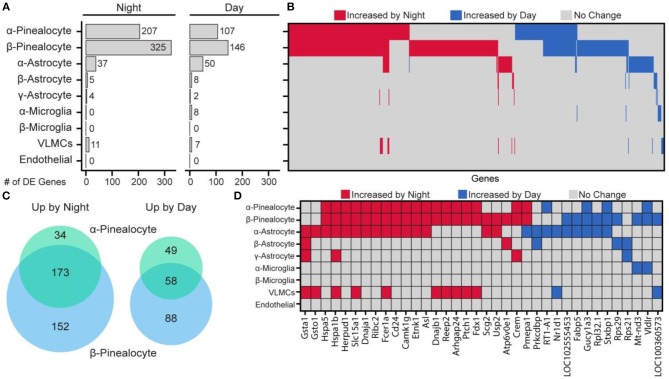
Changes in gene expression between day and night occur in a cell type-specific manner. **(A)** Number of differentially expressed (DE) genes upregulated by night or day by cell type. DE is *p* < 0.01 (Wilcoxon rank sum), when expressed in at least 15% of cells in either of the two samples being tested, fold change ≥ 2.0, and effect size ≥ 0.35. **(B)** Heatmap summary of all 644 DE gene changes by cell type. Each column represents one gene. **(C)** Venn diagram of number of overlapping DE genes in α- and β-pinealocytes by day and night. **(D)** Heatmap summary of DE genes found in at least one non-pinealocyte and one other subtype. See also dot plots in SI of Mays et al. (43). Reproduced from Mays et al. (43). This figure and associated legend is published with permission of the original publisher under license CC0.1.0.

Day and night expression values in specific pineal cell types were compared. There were considerable differences among the cell subtypes in the number of genes that were differentially expressed between day and night (Figure 6). The largest differential expression was found in pinealocytes: 359 genes were upregulated at night and 195 genes were upregulated during daytime. Consistent with prior studies, differentially upregulated genes included *Aanat, Crem, Drd4, Pde10a* (19, 25). Overall, β-pinealocytes had 1.5-fold more genes differentially expressed than α-pinealocytes, with considerable overlap: 173 and 58 of the same transcripts were increased in both subtypes during night and day, respectively (Figure 6C). Non-pinealocytes had generally lower day/night differential expression, with the α-astrocytes having 37 genes higher at night and 50 higher during the day. There were relatively fewer differentially expressed transcripts in other non-pinealocytes several of which overlapped between different cell types (Figure 6D). The molecular basis of changes in astrocytes is not clear. Whereas, α- and β-adrenergic mechanisms control changes in pinealocytes, the responsible receptors are absent from astrocytes. Other receptors might mediate these changes. Alternatively, the day/night differences could reflect the functioning of an internal clock in these cells, although expression of clock genes is not high [Figure S22 in (43)].

Pinealocytes have a high amount of *Aanat* at night. *Aanat* transcripts were also detected at uniformly low levels in non-pinealocytes [Figure S1 in (43)], probably due to contamination by pinealocyte-derived ambient mRNA. This results in non-pinealocytes erroneously seeming to express *Aanat* differentially. Because of this, the gene was deleted from non-pinealocyte analysis. *Pmepa1* was determined to be unusual because it was upregulated at night in one cell type (α- and β-pinealocytes) but upregulated during the day in α-astrocytes (Figure 6D).

## Isoproterenol Treatment Mimics Day/Night Changes in the Pineal Cell Transcriptomes of Pinealocytes

It is known that treatment with the β-adrenergic agonist isoproterenol during daytime has similar effects on the pineal transcriptome to those that occur due to neural stimulation at night. It is used in place of norepinephrine because isoproterenol is not taken up into nerve endings in the pineal perivascular space, whereas norepinephrine is rapidly and selectively taken up, thereby largely preventing adrenergic activation (54).

**Figure 7:**
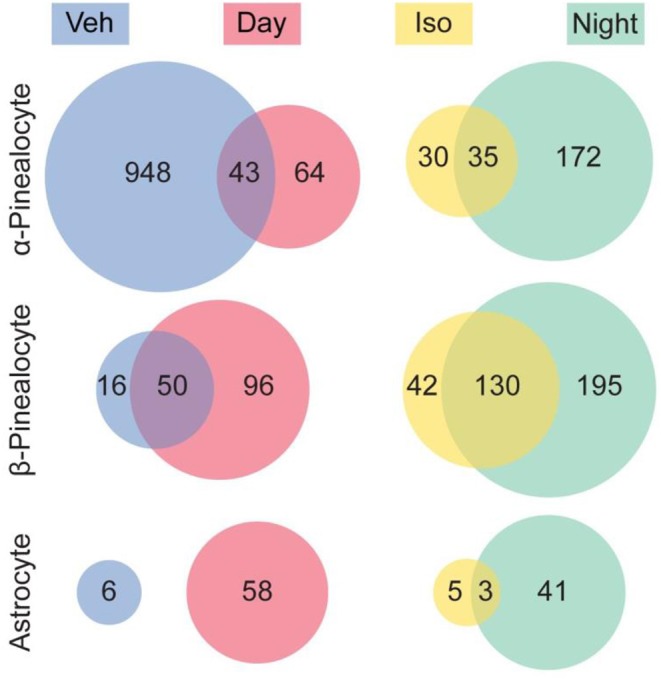
Comparison of differentially expressed genes between the night time pineal gland and isoproterenol-treated pineal gland. Venn diagrams indicate the number of genes that were significantly differentially expressed (DE) in the pineal gland. There was overlap between genes exhibiting DE upregulated at night and by isoproterenol (Iso) treatment, as well as overlap between genes exhibiting DE upregulated during the day and upregulated in the vehicle control treated (i.e., downregulated by isoproterenol treatment), in 3 cell types. Other cell types are not shown. Reproduced from Mays et al. (43). This figure and associated legend is published with permission of the original publisher under license CC0.1.0.

The results of our studies were in line with the interpretation that 97% of the transcriptional changes observed following isoproterenol treatment were in α- and β-pinealocytes (Figure 7). This is in agreement with findings of high enrichment with β-adrenergic receptors that mediate night time changes in gene expression. Astrocytes had the remaining 3% of changes, whereas changes in other cells were not detected. It should be noted that upregulation of a similar number of the same genes was observed in isoproterenol-treated glands and in night time glands: upregulation of 54, 76, and 38% of the same genes was seen in α-pinealocytes, β-pinealocytes, and astrocytes, respectively. Four and seventy-six percent of genes suppressed due to isoproterenol treatment in α-pinealocytes and β-pinealocytes, respectively, were also suppressed during the day, that is, they appeared to be upregulated after vehicle control treatment.

## Implications

There are broad implications of the findings of scRNA-seq analysis. Several points of interest can be identified, including the selective mechanisms involved in astrocyte gene expression. However, the feature which is especially worthy of additional comment here is the finding of two pinealocyte subtypes. As discussed above, α-pinealocytes are characterized by high levels of *Asmt* and high levels of the mitochondrial genome, and low levels of protein synthesis transcripts and *Gngt1* and *Gngt2*, in contrast to the more abundant β-pinealocytes (Figure 4B). Together, they are responsible for the synthesis of melatonin in the pineal gland, with slightly different roles.

**Figure 8:**
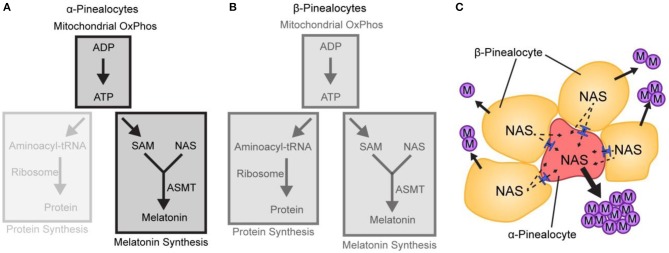
A hypothetical model based on scRNA-Seq depicting differences in melatonin synthesis between α- and β-pinealocytes. **(A,B)** The relative strength of a pathway module is indicated by opacity; greater opacity represents a more active pathway. **(A)** Conversion of N-acetylserotonin (NAS) to melatonin in α-pinealocytes is enhanced by higher ASMT activity and increased S-adenosyl methionine (SAM) availability, which is boosted by greater ATP availability. Increased ATP availability reflects increased ATP production from oxidative phosphorylation (OxPhos); this is inferred by greater expression of mitochondrial genes in α-pinealocytes. ATP availability also results from reduced consumption by protein synthesis, as inferred by decreased ribosomal transcriptome in α-pinealocytes. **(B)** β-Pinealocytes do not have the same enhancements as α-pinealocytes. **(C)** N-Acetylserotonin (NAS) that is not converted to melatonin in β-pinealocytes enters the α-pinealocyte by passive diffusion through membranes and gap junctions (shown in blue) and is converted to melatonin, thereby maximizing melatonin production. Reproduced from Mays et al. (43). This figure and associated legend is published with permission of the original publisher under license CC0.1.0.

It is proposed that the α-pinealocytes are especially highly adapted for the last step in melatonin synthesis. This is supported not only by the high levels of *Asmt* but by the accompanying increase in ATP production by the OxPhos pathway. The main impact on melatonin synthesis is that high ATP leads directly to an increase in SAM, which is synthesized from ATP and methionine (Figure 8). Thus, the cells containing both these effects are in a position to methylate N-acetylserotonin at high levels. The focus of the cells on melatonin synthesis is further evidenced by the low levels of protein synthesis enzymes; protein synthesis is the primary consumer of cellular ATP. Adding to this are the low levels of *Gngt1* and *Gngt2*, indicating that G-protein based signal transduction is suppressed; this is in agreement with the finding of lower levels of gene induction in these cells, as discussed above.

The existence of two functionally different pinealocyte subtypes raises the issue of whether α-pinealocytes are compromised to a degree that interferes with the functioning of these cells. This could occur due to relatively lower metabolites and suppressed protein synthesis. One can argue that the absence of some functions, such as maintenance of extracellular matrix, would be compensated for by β-pinealocytes. Also, some essential factors that are reduced in the α-pinealocytes could be provided by the β-pinealocyte. Cell:cell transfer of these factors via gap junctions, membrane permeability, and import/export mechanisms might mediate this export:import function. Also, some proteins may have sufficient stability to prevent a loss of function. Moreover, lowered activity of some processes in the α-pinealocytes may enhance ASMT activity by lowering the production of inhibitors and producing a more favorable biochemical environment for ASMT. Hence, it seems possible that such changes could support the seemingly compromised α-pinealocytes.

A final issue to be addressed is how the number of each of the pinealocyte subtypes is regulated. One hypothetical possibility is that distinct phenotypes develop early in ontogeny and the cells cannot undergo a shift from one subtype to another. In this scenario, the phenotypes are not reversible; their relative abundance might only reflect selective cell death and replacement. A second hypothetical possibility is that α- and β-pinealocyte phenotypes are reversible and can shift back and forth at any time during development and maturity. The controlling factor or factors could be circulating in nature or reflect neural stimulation, perhaps influenced by the day length. This hypothetical reversible mechanism might fine tune melatonin production.

## Concluding Comment

The work reviewed here is impressive in documenting how methods have evolved from requiring a few milligrams of tissue to document a single transcript to documenting thousands of transcripts in a single cell!

scRNA-seq establishes a new foundation for research on pineal cell biology by introducing new methods and concepts and by segregating gene expression into separate cells. Moreover, it has reshaped our thinking about the pineal gland by adding to the complex nature of the tissue, by providing transcriptionally defined cell types. The work is unique in that two states of physiological activity—day and night—are characterized, which adds another dimension to the value of scRNA-seq of this tissue.

Work on the pineal gland has the potential to improve our understanding of the basic mechanisms that underlie the function of this tissue in non-human primates and humans. Bulk sequencing of the rhesus pineal gland indicates that there are fundamental differences between it and the rat, as regards day/night changes in transcript abundance (22, 55). It will be of interest to use scRNA-seq technology to learn more about the human and rhesus pineal glands, with the intention of understanding how cells in this tissue communicate and are regulated.

One avenue that will challenge investigators is the analysis of isoform regulation (23) on a single cell basis, with the goal of understanding the association of specific isoforms with cell types and how they are regulated. The discoveries revealed by scRNA-seq and advanced forms of sequencing will shape future studies on pineal cell biology.

## Author Contributions

SC, CF, SH, LH, JMa, MCK, MWK, JMu, MR, LS, and DK: substantial contributions to the conception or design of the work, or the acquisition, analysis, or interpretation of data for the work. SC, SH, LH, JMu, MCK, MWK, JMa, MR, and DK: drafting the work or revising it critically for important intellectual content. SC, LH, SH, MWK, JMa, MR, JMu, MCK, MR, and DK: provided approval for publication of the content.

### Conflict of Interest Statement

The authors declare that the research was conducted in the absence of any commercial or financial relationships that could be construed as a potential conflict of interest.
